# Repetitive, but Not Single, Mild Blast TBI Causes Persistent Neurological Impairments and Selective Cortical Neuronal Loss in Rats

**DOI:** 10.3390/brainsci13091298

**Published:** 2023-09-08

**Authors:** Rita Campos-Pires, Bee Eng Ong, Mariia Koziakova, Eszter Ujvari, Isobel Fuller, Charlotte Boyles, Valerie Sun, Andy Ko, Daniel Pap, Matthew Lee, Lauren Gomes, Kate Gallagher, Peter F. Mahoney, Robert Dickinson

**Affiliations:** 1 Anaesthetics, Pain Medicine and Intensive Care Division, Department of Surgery and Cancer, Imperial College London, London SW7 2AZ, UK; 2 Royal British Legion Centre for Blast Injury Studies, Imperial College London, London SW7 2AZ, UK; 3 Department of Bioengineering, Imperial College London, London SW7 2AZ, UK

**Keywords:** blast traumatic brain injury, blast neurotrauma, blast trauma, functional deficits, repetitive brain injury, concussion

## Abstract

Exposure to repeated mild blast traumatic brain injury (mbTBI) is common in combat soldiers and the training of Special Forces. Evidence suggests that repeated exposure to a mild or subthreshold blast can cause serious and long-lasting impairments, but the mechanisms causing these symptoms are unclear. In this study, we characterise the effects of single and tightly coupled repeated mbTBI in Sprague–Dawley rats exposed to shockwaves generated using a shock tube. The primary outcomes are functional neurologic function (unconsciousness, neuroscore, weight loss, and RotaRod performance) and neuronal density in brain regions associated with sensorimotor function. Exposure to a single shockwave does not result in functional impairments or histologic injury, which is consistent with a mild or subthreshold injury. In contrast, exposure to three tightly coupled shockwaves results in unconsciousness, along with persistent neurologic impairments. Significant neuronal loss following repeated blast was observed in the motor cortex, somatosensory cortex, auditory cortex, and amygdala. Neuronal loss was not accompanied by changes in astrocyte reactivity. Our study identifies specific brain regions particularly sensitive to repeated mbTBI. The reasons for this sensitivity may include exposure to less attenuated shockwaves or proximity to tissue density transitions, and this merits further investigation. Our novel model will be useful in elucidating the mechanisms of sensitisation to injury, the temporal window of sensitivity and the evaluation of new treatments.

## 1. Introduction

Traumatic brain injury (TBI) is a major cause of mortality and morbidity in both military and civilian populations [1,2,3,4]. In the last 22 years, more than 470,000 US service personnel have been diagnosed with a TBI [5]. Exposure to blasts, particularly from improvised explosive devices (IEDs), has become a significant cause of military TBI [6,7], and blast TBI has been called a “signature injury” associated with military operations in Iraq and Afghanistan [8]. Blast TBI is increasingly experienced by civilians in conflict zones [9] and during terrorist attacks or industrial explosions. The 2020 Beirut port explosion, which was the largest non-nuclear explosion in recent history, was caused by the detonation of 2750 tons of inappropriately stored ammonium nitrate fertilizer; this explosion, occurring in an urban environment, resulted in over 6000 civilian injuries and 200 fatalities [10]. A multicentre study of the injury characteristics of patients presenting at emergency departments after the Beirut explosion found that 6.2% had concussion originating from primary blast exposure [11]. The current (2022) conflict in Ukraine has seen cities and civilian infrastructure targeted by artillery and guided weapons [12].

The majority (~80%) of TBIs in both military and civilian populations are mild [4,5,7]. Due in part to an overlap of symptoms with other conditions, mild blast TBI can be difficult to diagnose. A particular concern in military deployment and training is exposure of personnel to repeated mild blast injuries and concussion [13]. In addition to exposure to multiple mild blasts from IEDs during operations, there are specific groups, such as breachers and artillery personnel, that are routinely exposed to multiple mild blasts [14]. Repetitive head injury is also common in contact sports, such as boxing, American football, and rugby, as well as in ‘non-contact’ association football [15,16,17]. It is estimated that sports-related injuries in the USA annually result in up to 3.8 million concussions, but this number may be significantly higher due to under reporting [16]. 

Historically, there has been controversy regarding whether mild or subconcussive head injuries can have lasting effects. However, it is now recognised that exposure to repeated mild subthreshold TBI (which, when considered alone, results in minimal or no effect) can lead to lasting cognitive and behavioural impairments and increases the risk of patients developing chronic traumatic encephalopathy (CTE) and other neurodegenerative conditions [14,17,18,19,20,21]. The mechanisms underlying the factors leading initial subthreshold exposure to cause sensitisation of the brain to subsequent injury remain unclear, as is the answer to the question of whether specific brain areas are more sensitive to repetitive injury. 

Real-life blast neurotrauma consists of a number of components: primary blast injury resulting from the blast shockwave, secondary blast injury resulting from blunt or penetrating injury caused by projectiles or fragments, and tertiary blast injury resulting from acceleration/deceleration due to inertial displacement [22,23,24]. While blunt and penetrating brain trauma have been more widely studied, the other components of blast neurotrauma are much less well understood, and consensus is lacking as to the way in which primary and tertiary blast injury mechanisms interact to cause this pathology [19,25,26,27]. 

Animal models have been playing an important role in understanding blast neuropathology, and attention has focused on the blast shockwave, which is unique to blast neurotrauma. A large number of animal studies investigating moderate-to-severe blast TBI resulting from exposure to a single blast shockwave have reported behavioural deficits and neuropathological changes (for a review, see [28]). Several pre-clinical studies provided evidence of an acute neuroglial inflammatory response following blast [19,25,27,29,30]. In contrast, with some notable exceptions [31,32,33,34], few early studies investigated the effects of repeated blast TBI. However, in recent years, interest in the effects of repetitive mild blast TBI has increased, with studies demonstrating behavioural deficits and neuroinflammation [35,36,37]. Whether the inflammatory response leads directly to loss of neurons is as yet unclear, but reactive astrogliosis has been observed in the brains of human blast victims [18,19,38], and serum biomarkers of neuronal injury have been detected in personnel exposed to blasts [39,40]. 

Our aim was to investigate and characterise the effects of single and repeated mild blast-induced TBI in a novel rat model incorporating primary and tertiary blast components, as well as to determine whether repeated mild blasts result in behavioural deficits, neuronal loss, and astrogliosis. Primary outcomes were neurologic and motor function and neuronal density in the associated brain areas. Secondary outcomes were neuronal density in other regions of interest and astrogliosis.

## 2. Materials and Methods

### 2.1. Experimental Subjects

Experiments were performed in compliance with the UK Animals (Scientific Procedures) Act of 1986 and approved by the Animal Welfare and Ethical Review Body of Imperial College London (PPL 7007466/PD8ED7926). Our study design complies with the ARRIVE and PREPARE guidelines [41,42]. Young adult male Sprague–Dawley rats (n = 90) aged 10–12 weeks old were obtained from Charles River Laboratory (Margate, UK). Animals had undergone no previous procedures before entering the study. Animals were housed in groups of 3–4 in filter-top cages in a pathogen-free facility in a 12:12 light–dark cycle (7 am–7 pm light) at 22 °C with ad libitum access to food and water. Animals were monitored daily before experiments, continuously monitored in the post-blast period for at least 4 h, monitored again early the following day and then daily until the end of the experimental protocol. 

### 2.2. Experimental Groups, Randomizing, and Blinding

Animals were randomly assigned to sham or blast groups using a computerised random number generator. The distributions of animals were 1 × blast (n = 22), 1 × sham (n = 18), 3 × blast (n = 28), and 3 × sham (n = 22). Animals were allowed to survive for 24 h, 5 d, or 22 d. A subset of these animals was used to perform histological analysis at 24 h and 5 d. Planned group sizes for the histological outcomes were 6–8 animals per group, with this size based on early pilot studies. The actual histological group sizes were 1 × blast 24 h (n = 8), 1 × sham 24 h (n = 8), 1 × blast 5 d (n = 9), 1 × sham 5 d (n = 8), 3 × blast 24 h (n = 7), 3 × sham 24 h (n = 6), 3 × blast 5 d (n = 8), and 3 × sham 5 d (n = 6). The blast procedure, including animal preparation and the operation of the shock tube, was carried out by one experimenter. A separate experimenter, who was blinded to the groups, performed neurologic tests. Histological outcomes were assessed by blinded observers. 

### 2.3. Blast TBI Procedure and Neurological Outcomes

**Figure 1:**
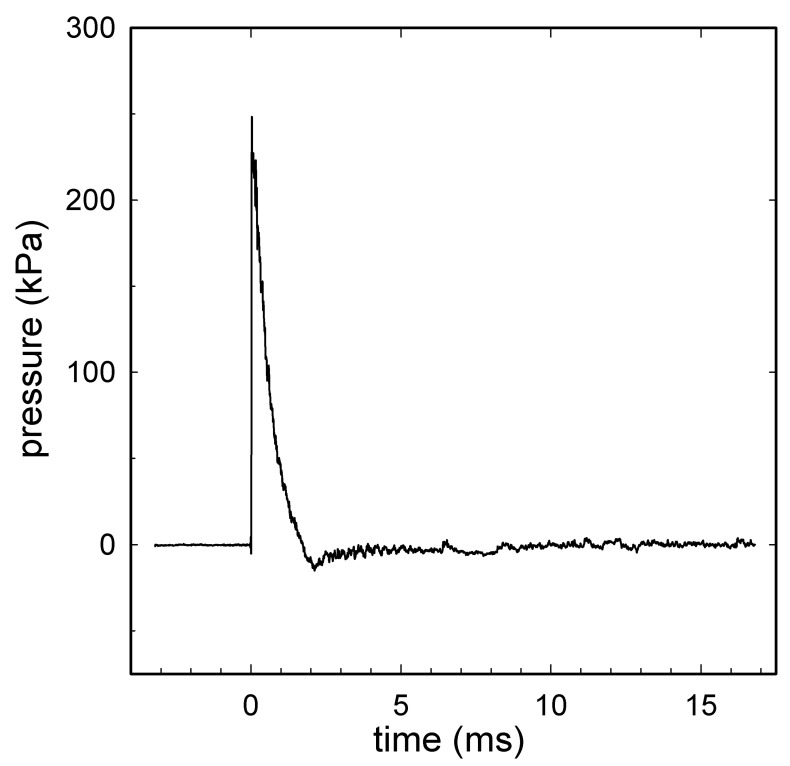
A representative shock wave recorded during the experiment with animal in position. A signal obtained from a radial pressure sensor (2300 V1, Dytran Instruments, Chatsworth, CA, USA) at the distal end of the shock tube: the radial peak overpressure was 246 kPa; the positive wave duration was 1.3 ms; the impulse was 117 kPa·ms. Compressed air was used in the shock tube with a single diaphragm configuration using 225 µm Mylar^®^. Data were recorded using a high-bandwidth oscilloscope (Tektronix model DPO4104B Tektronix Inc., Beaverton, OR, USA) at a sampling rate of 50 MHz, before being digitally filtered offline at 40 kHz.

A shock tube blast generator developed for use in a variety of *in vitro* and *in vivo* biological models, described previously [43,44,45,46,47,48,49,50] was used to generate shockwaves with a Friedlander waveform (Figure 1). Shockwave data were obtained during the experiments from a radial pressure sensor at the distal end of shock tube [48] with animals in position. 

Animals were anaesthetised with propofol (*via* a tail vein cannula) through a series of bolus injections in order to maintain an appropriate depth of anaesthesia, which was evaluated *via* repeated assessment of the lack of a pedal withdrawal reflex. The amount and timing of propofol administration were recorded and were similar for blast and sham groups in each configuration. Analgesia was provided *via* subcutaneous injection of buprenorphine before blast exposure or sham procedure (0.03 mg/kg or 0.04 mg/kg for single or repeated blast respectively). The sham and blast groups for each blast configuration received the same dose of propofol (mg/kg) (Supplementary Table S1). 

**Figure 2:**
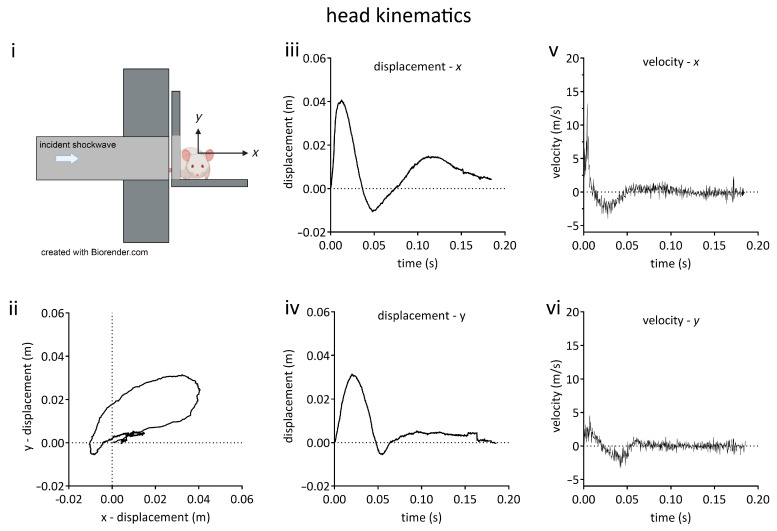
Kinematic characterisation of rat head movement in the blast neurotrauma model. (**i**) Schematic diagram of animal orientation and positioning on platform at distal end of shock tube. Rats were oriented with the right side of the head towards incident shockwave, being positioned such that only the head was exposed and the thorax was shielded from the shockwave. The head could move unrestrained in the x-y plane. (**ii**) Head displacement in x-y plane. (**iii**) Head x-displacement as a function of time. (**iv**) Head y-displacement as a function of time. (**v**) Velocity in x axis as a function of time. (**vi**) Velocity in y axis as a function of time. Lines shown are the means of data obtained from 3 animals.

Animals were placed onto a horizontal stainless steel platform with a 10-millimetre-thick Sorbothane sheet (KM Sounds Ltd., Staines-Upon-Thames, UK) bolted to the distal end of the shock tube. Animals were positioned such that the head was the only part of the body exposed to the shockwave, and they were secured in position inside of a custom-made tight-fitting plastic restraining cone attached to the platform using Velcro^®^ straps held in place with metal clamps. The centre of the cranium was aligned with the vertical axis of the shock tube outlet, being as close as possible to the shock tube (head midline 2 cm from outlet), perpendicular to the shock tube, with the right side of the head facing the outlet (Figure 2).

The thorax and abdomen were positioned along the outlet flange such that they were shielded from the shockwave, avoiding blast-lung or thoracic systemic inflammatory response. Due to the securing of the rat body, any motion along the longitudinal axis of the body (*z*-axis) was constrained. Sham animals were positioned on the platform for the same amount of time as the blast animals, but the shock tube was not fired. All animals were given supplemental oxygen (0.5 L/min) and were spontaneously breathing throughout the procedure. Animals were exposed to either a single shockwave, with peak overpressure, duration, and impulse of 259 ± 3 kPa, 1.40 ± 0.10 ms and 132 ± 8 kPa·ms, respectively, or to three tightly coupled shockwaves, with peak overpressure, duration, and impulse of 256 ± 2 kPa, 1.30 ± 0.03 ms, and 129 ± 3 kPa·ms, respectively, with an inter-blast interval of 7.0 ± 0.2 min, which were similar to the previously described models of repetitive blast injury [31,51,52].

### 2.4. Physiologic Monitoring

Physiological monitoring in animal models of brain injury mirrors the processes that happen in human trauma patients and is recognised as part of good experimental design [53,54,55]. During anaesthesia induction and recovery, animals were placed onto a temperature-controlled heating mat and given supplemental oxygen (0.5 L/min). Clinically relevant physiologic parameters were monitored, including peripheral blood oxygenation *via* pulse oximetry (OXY-100 Vet Pulse Oximeter, Gima, Gessate, Italy), non-invasive systolic and diastolic blood pressure, heart rate, and core body temperature (Kent CODA monitor, EMKA Technologies, Paris, France). 

### 2.5. High Speed Videography and Kinematic Analysis

A Phantom v611 high speed video camera (Vision Research Inc., Wayne, NJ, USA) with a frame rate of 100,000 fps was positioned facing towards the nose of the rat placed onto the platform at the distal end of the shock tube. Videos were acquired using Phantom Camera Control v3.5 software (Vision Research Inc., Wayne, NJ, USA) and stored on a computer.

To provide reference points, under anaesthesia, two lines were drawn using a marker pen, with one line horizontally drawn across the head at a point between the ears and another perpendicular line drawn from a point midway between the ears and the nose. The reference point for the kinematic analysis was the point midway between the ears at which the line from the nose intersected the horizontal line. The origin of the x-y plane (Figure 2i) in each video was the position of the intersection between these two lines in the frame before movement began. The position of this reference point was tracked until the head movement ended. Data regarding x- and y-positions and velocities were calculated for each animal using Tracker v5.1.5 software (Open Source Physics, www.compadre.org), with sampling occurring every 20 frames (0.2 ms). The regions of maximum change in velocity were determined by inspection of the x- and y-velocity versus time graphs, and maximum accelerations were calculated from a linear fit of tangents to these graphs at these points.

### 2.6. Blast-Induced Unconsciousness: Loss of Righting Reflex

The increase in the time until the recovery of the righting reflex after the blast compared to sham animals was used as a measure of the blast-induced loss of consciousness [56]. After blast exposure, the animals were placed in the supine position on a feedback-controlled temperature mat until they righted themselves (all four paws placed on the surface of the mat). The time from the last shock wave until the recovery of the righting reflex was recorded [56]. Following the recovery of the righting reflex, animals were returned to their home cages.

### 2.7. Neurological Outcome Score

We used a modified version of a neurological outcome score [57]. Our modified version focused on simple and complex motor function, including beam walking (2.5 cm beam) and general status; we omitted sensory tests. Uninjured healthy animals should score zero, with severely impaired rats scoring a maximum of 34 points. We assessed the baseline neurological score 1 day before the procedures and again 1 day after the blast or sham procedure.

### 2.8. Vestibulomotor Function

Vestibulomotor function was assessed using a RotaRod (model 47700, Ugo Basile, Gemonio, Italy). Prior to the procedures, animals underwent three days of training to achieve steady baseline. On day 0 of training, animals underwent acclimatisation to the Rotarod at a constant low speed of 4 rpm for 5 min. On training days 1 and 2, an accelerating protocol was used, starting at 4 rpm with a linear increase in speed up to 40 rpm over the 5-minute trial period. Each animal underwent 3 consecutive trials, with 5 min between trials. 

The latency to fall was recorded using the single best trial (longest time) as a measure of performance, with testing occurring using the accelerating protocol at 1 day, 5 days, 15 days, and 22 days after the procedure. 

### 2.9. Body Weight Change

Animals were weighed on the day of the procedure (day 0). Animals’ body weights were monitored in the days following procedure, and the weight change for each animal was calculated as a percentage of its pre-procedure body weight.

### 2.10. Histological Processing, Immunofluorescence Staining, Imaging, and Analysis

Histological processing, immunofluorescence staining, and imaging procedures were similar to those previously described [58]. At each histology endpoint, animals were terminally anaesthetised using intraperitoneal pentobarbital and transcardially perfused using ice cold phosphate buffered saline (PBS) (Sigma Aldrich, Gillingham, Dorset, UK), followed by ice cold 4% paraformaldehyde (Thermo-Fisher Scientific, Loughborough, UK) in PBS. 

The brains were carefully removed from the skull and post-fixed in 4% paraformaldehyde in PBS overnight at 4 °C, before being transferred to 30% sucrose (Sigma Aldrich, Gillingham, Dorset, UK) solution in PBS until equilibration. Brains were frozen in powdered dry ice and stored at −80 °C until processing. Frozen brains were embedded in optimal cutting temperature medium (OCT, Thermo-Fisher Scientific, Loughborough, UK), and 20-micrometre coronal sections were cut using a cryostat (Leica CM3050, Leica Microsystems, Milton Keynes, UK). 

Slices were stained with antibodies of neuronal nuclei (NeuN), glial fibrillary acid protein (GFAP), and the nuclear stain DAPI, as previously described [58]. Images were captured using a Zeiss AxioObserver Inverted Widefield Microscope (Facility for Imaging by Light Microscopy, Imperial College London) equipped with a motorised stage and a 20 × objective (Zeiss Plan Apochromat, NA 0.8, WD 0.55 mm), as described previously [58]. 

### 2.11. Image Analysis

**Figure 3:**
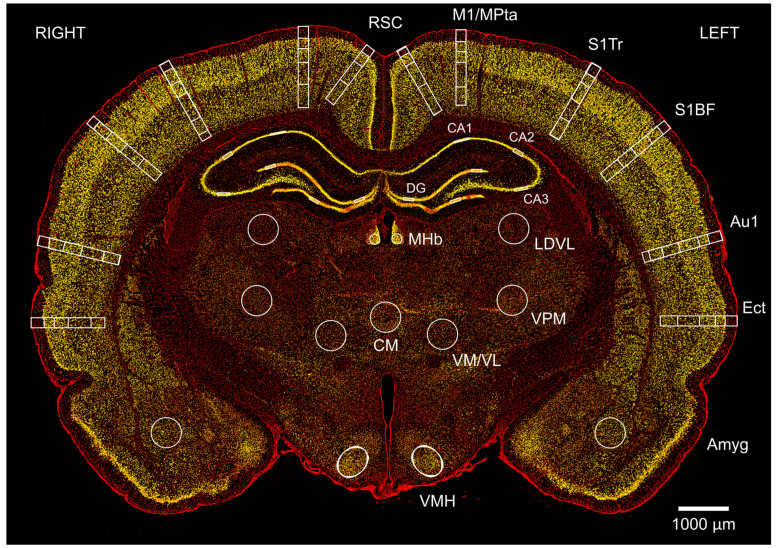
Changes in neuronal density after single and repeated blast injury were mapped in cortical and subcortical regions in coronal brain sections. Image shows a typical section at Bregma −3.36 mm from a sham animal at 24 h, stained with neuronal marker NeuN (yellow) and non-specific nuclear marker DAPI (red). Neurons were counted in left and right retrosplenial cortex (RSC) in layers 1, 2/3/4, and 5/6; the motor/medial parietal association cortex (M1/MPtA) was assessed in layers 1, 2/3, 4, and 5/6; the somatosensory cortex trunk region and barrel field (S1Tr, S1BF) were assessed in layers 1, 2/3, 4, and 5/6; the auditory cortex (Au1) was assessed in layers 1, 2/3, 4, and 5/6; and the ectorhinal cortex (Ect) was assessed in layers 1, 2/3, 4, and 5/6. Finally, in subcortical regions of amygdala (Amyg), ventromedial hypothalamus (VMH), centromedial thalamic nucleus (CM), ventromedial/ventrolateral thalamic nucleus (VM/VL), ventral posteromedial thalamic nucleus (VPM), laterodorsal thalamic nucleus, ventrolateral (LDVL), and medial habenular nucleus (MHb) and hippocampus (CA1; CA2; CA3 and DG) were assessed. The scale bar is 1000 μm.

NeuN positive neurons were manually counted using Fiji (ImageJ) [59], as described previously [58]. Figure 3 shows a typical slice stained with NeuN and DAPI showing the neuronal regions of interest (ROIs). Our injury paradigm had the right side of the head perpendicular to the shockwave, and there was a possibility of a lateralisation of the neuronal loss; therefore, we quantified the neuronal density in both left and right hemispheres. Astrocyte activation was assessed by measuring the percentage GFAP positive area, as described previously [58]. Similar imaging parameters (exposure time and LED intensity) were used to acquire the images, and the same binarisation threshold was used for all images in the GFAP area analysis. The dimensions of the ROIs used are described in the Supplementary Information document. 

### 2.12. Lung Tissue Processing and Histology

Following euthanasia under anaesthesia, fresh lungs were extracted from the thoracic cavity and inflated with approximately 6 mL of PBS and trachea tied off. The samples were placed in individual 50-millilitre tubes containing neutral buffered formalin. After 48 h, formalin was replaced with 70% ethanol. Samples were stored in the dark at 4 °C until further processing. The right caudal lobe was removed, placed in a biopsy cassette containing 70% ethanol, and embedded in paraffin wax prior to cutting. Slices (5 μm) were cut using a Leica rotary microtome (model 2125RT, Leica Microsystems, Milton Keynes, UK) and stained with mercury-free haematoxylin and eosin (H&E) (VWR Ltd., Leicester, UK). A Zeiss AxioObserver widefield microscope with 20 × objective (Zeiss Plan Apochromat, NA 0.8, WD 0.55 mm) was used to perform brightfield imaging.

### 2.13. Statistics

For the loss of the righting reflex, propofol dose, neurologic outcome score, and immunohistology results, the blast groups and sham groups were compared using a Mann–Whitney test or a Kruskal–Wallis test with Benjamini–Yekutieli correction. 

Rotarod and body weight change data were analysed using a two-way ANOVA with Sidak’s correction. *p* values of less than 0.05 were taken to indicate a significant difference between groups. Unless otherwise stated, values are quoted as means ± SEM. Sample sizes, designated as n = number of animals, are indicated in the figure legends. Statistical tests were implemented using GraphPad Prism version 7.03 for Windows (GraphPad Software, GraphPad Software, Boston, Massachusetts, USA, www.graphpad.com (accessed on 30 July 2023)).

## 3. Results

### 3.1. Characterisation of Head Kinematics

Head motion in the x-y plane (Figure 2) described an oval trajectory, following exposure to a 260-kilopascal shockwave. The kinematic parameters, x-y displacements, velocities, and accelerations are given in Table 1. Motion along the longitudinal axis of the body (*z*-axis) was prevented (see Methods). 

### 3.2. Exposure to Single Blast Shockwave Does Not Affect Physiological Parameters

Peripheral oxygen saturations were in the normal range (median values 95% or above; minimum values of ≥90%) before and after the blast (Supplementary Table S1). Core body temperature, heart rate, and blood pressure (systolic and diastolic) were within the normal physiological range before and after the blast, with no significant differences in any parameters recorded before and after the procedures or between the sham and blast groups (Supplementary Table S1). 

### 3.3. Exposure to Single Blast Shockwave Does Not Result in Functional Impairments

**Figure 4:**
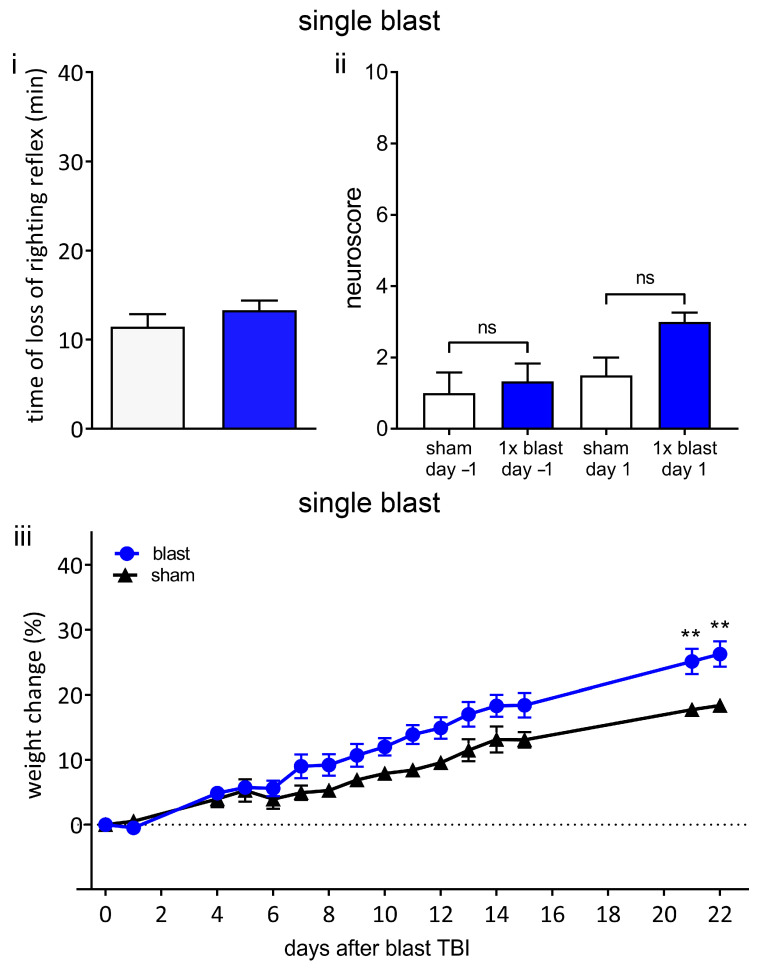
Single blast does not result in unconsciousness or persistent neurological deficits. (**i**) A single 260-kilopascal shockwave does not result in blast-associated unconsciousness. Time until recovery of righting reflex immediately after blast (blue bar) was not significantly different to that of sham procedure (white bar), indicating that there was no blast-associated unconsciousness. (**ii**) Neurological outcome score in blast and sham groups was not significantly different one day before blast or one day after blast. (**iii**) Weight change relative to procedure day up to 22 days following blast or sham procedure. There was no weight loss in either sham or blast group. Values shown are means, and error bars are standard errors. ** *p* < 0.01, ns not significant. Loss of righting reflex, 1 × blast n = 22, sham n = 18, Mann–Whitney test. Neuroscore 1 × blast n = 6, sham n = 4, Kruskal–Wallis test with Benjamini–Yekutieli correction. Weight change, 1 × blast n = 16, sham n = 10, two-way ANOVA with Sidak’s correction.

There was no significant difference in the time required to recover the righting reflex, as a measure of blast-induced unconsciousness, between single blast group (13.3 ± 1.1 min) and the sham group (11.4 ± 1.4 min) (Figure 4i). There was no difference in the dose of propofol administered to each of the two groups (Supplementary Table S1). These findings are consistent with a single blast of this intensity not inducing unconsciousness. There was no significant difference in the neurological outcome score between the single blast and the sham group before or after the blast or sham procedure (Figure 4ii). 

Weight loss in the days after TBI in rodents has been shown to correlate with neurological and sensorimotor deficits, injury severity, and neuronal loss [60,61]. The change in body weight compared to the day of the procedure is shown in Figure 4iii. There was no weight loss in either group after the blast or sham procedure. Compared to the sham group, the single blast group was not significantly different up to day 15, but percentage weight increases of the single blast group were significantly greater than those of the sham group at day 21 and day 22. These findings are consistent with this intensity of a single blast not resulting in global neurological or sensorimotor deficits.

### 3.4. Exposure to Single Blast Shockwave Does Not Result in Cortical Neuronal Loss

No significant differences in neuronal cell density between the single blast and sham groups were observed at either time point in any of the cortical areas investigated (Supplementary Figures S1 and S2i–iv). 

### 3.5. Exposure to Single Blast Shockwave Does Not Result in Subcortical Neuronal Loss

No significant differences in neuronal cell density between the single blast and sham groups were observed at either time point in any of the subcortical areas investigated (Supplementary Figure S2v–viii). 

Taken together, the lack of blast-induced unconsciousness after a single shockwave of 260 kPa, together with the lack of neurological impairments, or weight loss and the absence of cortical or subcortical neuronal loss are consistent with this intensity of a single blast resulting in a very mild, or subthreshold, blast TBI. 

### 3.6. Exposure to Three Repeated Blast Shockwaves Does Not Affect Physiological Parameters or Cause Lung Injury

**Figure 5:**
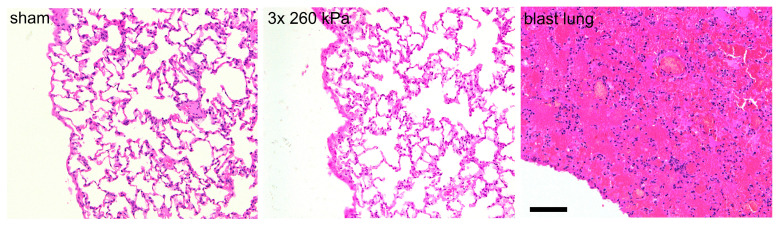
Head-only blast model avoids lung injury. Typical haematoxylin- and eosin-stained slices obtained from right caudal lobe of lungs of animals exposed to sham procedure or three repeated shockwaves at 24 h after the blast or sham procedure. Also shown is right caudal lobe obtained from an animal with fatal blast lung. The alveolar spaces in the sham and three repeated blast animals are clear, well inflated, and free of red blood cells. In contrast, the tissue obtained from animal with blast lung exhibits severe pulmonary haemorrhage, or ‘hepatisation’. Scale bar is 100 μm.

Peripheral oxygen saturations were found to be in the normal range (median values 97% or above; minimum values of ≥92%) before and after the blast (Supplementary Table S1). The normal oxygen saturations after the blast are consistent with good peri-procedure care, as well as with our configuration effectively shielding the thorax and lungs from the shockwave. To further investigate whether there was any pulmonary injury, we carried out a histological examination of the lung tissue. Lung tissues from sham and blast animals were normal and clearly distinguishable from blast lung (Figure 5). The core body temperature, heart rate, and blood pressure (systolic and diastolic) were within the normal physiological range before and after the blast (Supplementary Table S1). There were no significant differences in any of the parameters, except temperature, before and after the procedure or between the sham and repeated blast groups. In the case of core body temperature, there were no significant differences between the sham and repeated blast groups, but there was a significant reduction in the temperature (to 37 °C) in both groups after the blast or sham procedure (Supplementary Table S1). 

### 3.7. Exposure to Three Repeated Blast Shockwaves Results in Acute and Persistent Functional Deficits

**Figure 6:**
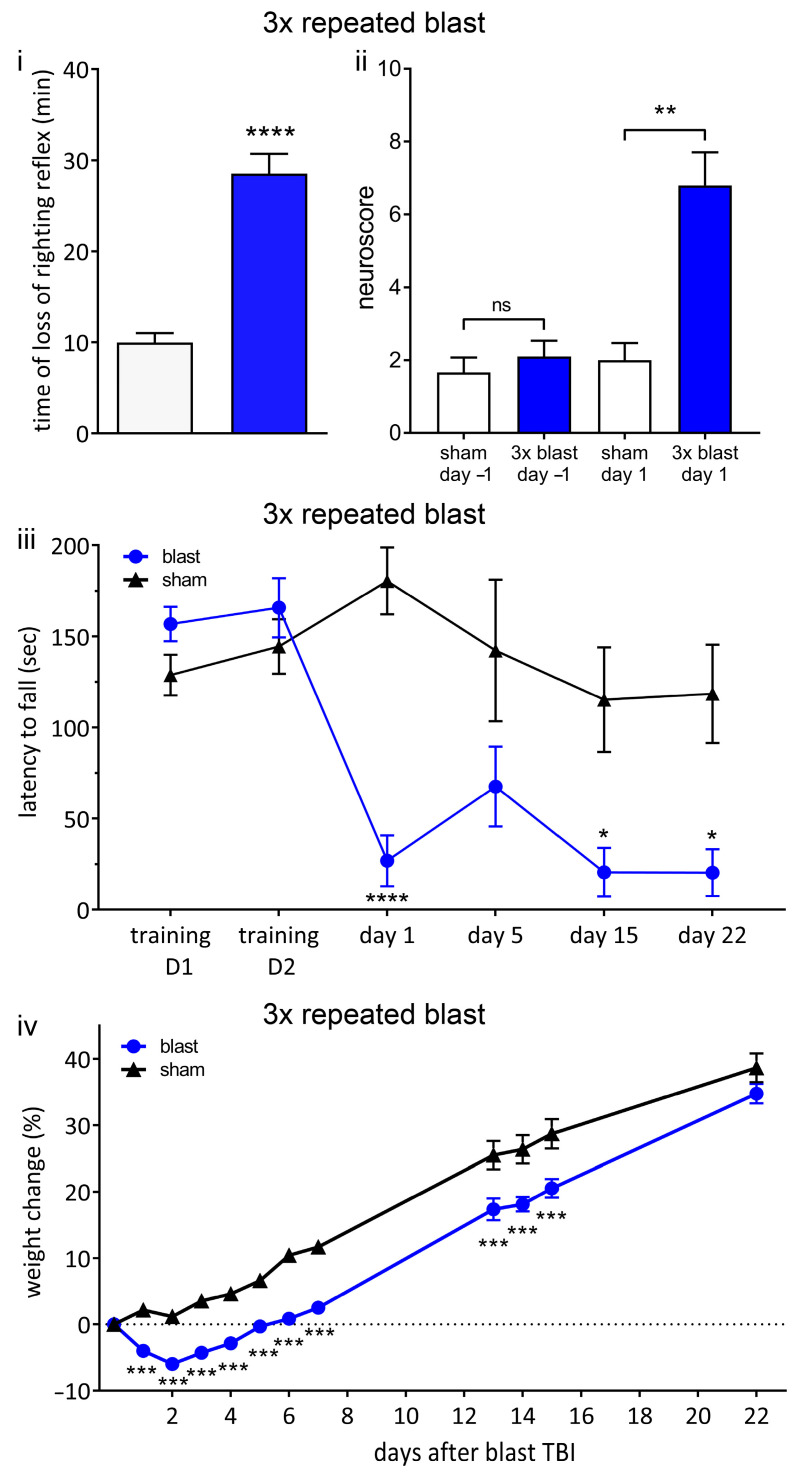
Repeated blast results in unconsciousness and persistent neurological deficits. (**i**) Three repeated shockwaves result in blast-associated unconsciousness. Time until recovery of righting reflex immediately after blast (blue bar) was significantly increased compared to sham procedure (white bar), indicating blast-induced unconsciousness. (**ii**) There are neurological impairments after three repeated shockwaves. One day before procedure, there was no significant difference between blast (blue bar) and sham (white bar) groups. Following blast, there was a significant increase in neuroscore in blast group compared to sham group, indicating neurological impairments. (**iii**) RotaRod performance indicates persistent vestibulomotor deficits following repeated blast exposure. There was no difference in latency to fall between blast and sham groups on two training days before injury. Latency to fall was significantly decreased in blast group on day 1, day 15, and day 22 after blast. (**iv**) Weight change relative to procedure day up to 22 days following blast or sham procedure. There was no weight loss in sham group. There was significant weight loss in repeated blast group compared to sham group. Values shown are means, and error bars are standard errors. * *p* < 0.05, ** *p* < 0.01 *** *p* < 0.001, **** *p* < 0.0001, ns not significant. Loss of righting reflex, 3 × blast n = 28, n = 22 sham, Mann–Whitney test. Neuroscore 3 × blast n = 10, sham n = 9, Kruskal–Wallis test with Benjamini–Yekutieli correction. Weight change, 3 × blast n = 25, sham, n = 22, two-way ANOVA with Sidak’s correction. Rotarod; 3 × blast n = 15, sham n = 12, two-way ANOVA with Sidak’s correction.

In contrast to a single blast, three repeated blasts resulted in prolonged and significant blast-induced unconsciousness (Figure 6). The time until the recovery of the righting reflex after the final blast was significantly increased (28.5 ± 2.2 min, *p* < 0.001) compared to the sham group (10.0 ± 1.0 min) (Figure 6i). There was no significant difference in the total dose of propofol (mg/g) between the sham and repeated blast groups (Supplementary Table S1).

One day following the blast, there were significant (*p* < 0.01) neurological impairments (increase in neuroscore) in the repeated blast group (6.8 ± 0.9) compared to the sham group (2.0 ± 0.5) (Figure 6ii). 

In order to assess vestibulomotor function, we measured latency to fall in an accelerating RotaRod protocol (Figure 6iii). Before the blast or sham procedure, there was no difference between groups in terms of RotaRod performance (training day 1, training day 2). In contrast, following repeated blast TBI, the latency to fall was significantly (*p* < 0.05) reduced compared to the sham group on day 1, day 15, and day 22, indicating a persistent locomotor deficit (Figure 6iii). On day 5, after repeated blasts, the latency to fall was reduced, but this reduction did not reach significance. These findings are consistent with prolonged vestibulomotor impairments following repeated blasts. 

The sham group exhibited an approximately linear increase in weight as a function of time, as expected for animals with ad libitum access to food and water (Figure 6iv). In contrast, the repeated blast group demonstrated weight loss on days 1 to 5 following the blast, returning to baseline weight only on day 6, followed by an approximately linear increase in weight as a function of time. The relative body weight in the repeated blast group was significantly (*p* < 0.001) less than that of the sham group at all time points, except day 22 (Figure 6iv). On day 22, the relative weight of the repeated blast group was less than that of the sham group, but the difference did not reach significance. 

Overall, these functional outcomes are consistent with repetitive mild blast resulting in acute blast-induced loss of consciousness and persistent neurological and vestibulomotor deficits.

### 3.8. Exposure to Three Repeated Blast Shockwaves Causes Cortical Neuronal Loss

**Figure 7:**
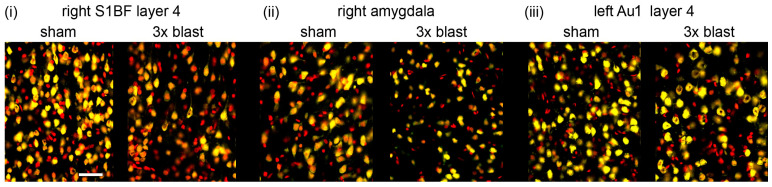
Repeated 260-kilopascal blast results in neuronal loss. Representative neuronal staining in sham and three repeated blast groups in (**i**) right somatosensory cortex (S1BF) layer 4, (**ii**) right amygdala, and (**iii**) left auditory cortex (Au1) layer 4. NeuN positive cells are shown in yellow, and DAPI is shown in red. Images represent areas included in neuronal quantification in Figure 7 and Figure 8. The scale bar is 50 μm.

**Figure 8:**
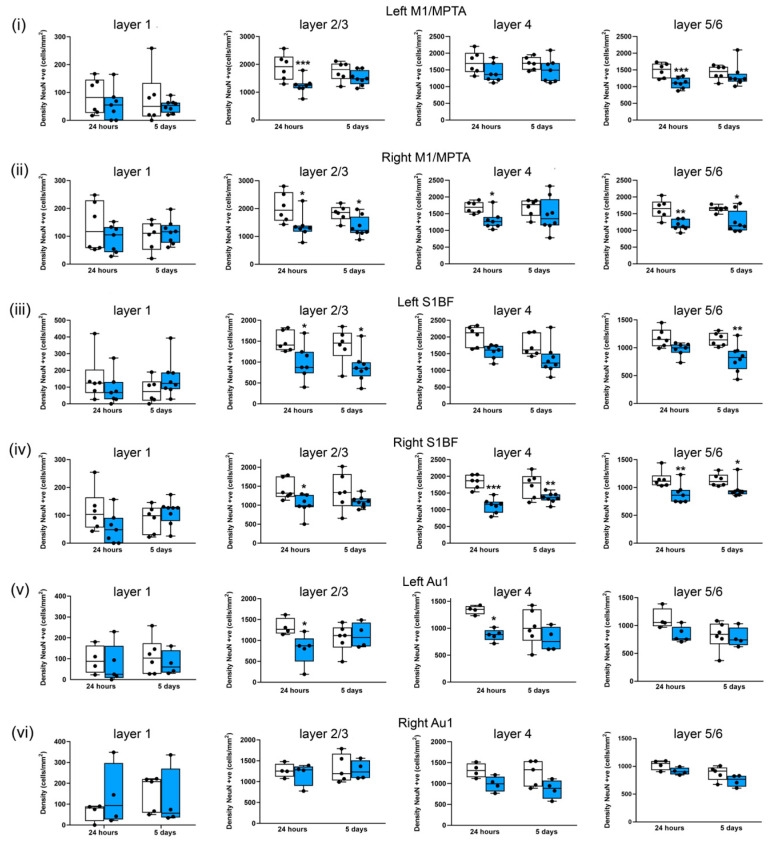
Three repeated shockwaves result in cortical neuronal loss. Quantification of neuronal cell density of cortical layers from sham (white boxes) and three repeated 260-kilopascal blasts (blue boxes) groups in (**i**) left and (**ii**) right motor/medial parietal association cortex (M1/MPtA), (**iii**) left and (**iv**) right somatosensory cortex (S1BF), and (**v**) left and (**vi**) right auditory cortex (Au1). Lines are medians, boxes represent interquartile intervals, and whiskers are ranges. * *p* < 0.05, ** *p* < 0.01, and *** *p* < 0.001 compared to sham; Kruskal–Wallis test with Benjamini–Yekutieli correction. After 24 h, sham n = 6 and blast n = 7; after 5 days, sham n = 6, and, blast n = 8. Not all slices included Au1.

Representative images of NeuN-stained sections showing the somatosensory cortex (S1BF), amygdala, and auditory cortex (Au1) from the sham and repeated blast groups are shown in Figure 7. There were significant reductions in the median neuronal densities in the left M1/MPta in layers 2/3 (by 35%, *p* < 0.001) and layers 5/6 (27%, *p* < 0.001) 24 h after the blast (Figure 8i). Similar reductions in median neuronal density were observed after 5 days, but these reductions did not reach significance. In the right M1/MPta, there were significant reductions in the median neuronal densities in layers 2/3 after both 24 h and 5 days (33%, *p* < 0.05 and 35%, *p* < 0.05 respectively), layer 4 after 24 h (25%; *p* < 0.05), and layer 5/6 after both 24 h and 5 days (33%, *p* < 0.01 and 31%, *p* < 0.05, respectively) (Figure 8ii). In layer 4, there was a reduction in the density of a similar magnitude at 5 days after the blast, but this reduction did not reach significance. In the left S1BF, there were significant reductions in the median neuronal density after repeated blasts in layers 2/3 after 24 h and 5 days (by 38%, *p* < 0.05 and 41%, *p* < 0.05 respectively) and layers 5/6 after 5 days (by 28%, *p* < 0.01) (Figure 8iii). Similar reductions in the median density were observed in layer 4 after 24 h and 5 days and layer 5/6 after 24 h, but these reductions did not reach significance. In the right S1BF, there were significant reductions in the median neuronal densities after repeated blasts in layers 2/3 after 24 h (by 25%, *p* < 0.05), layer 4 after 24 h and 5 days (by 38%, *p* < 0.001 and 25%, *p* < 0.01 respectively), and layer 5/6 after 24 h and 5 days (by 23%, *p* < 0.01 and 16%, *p* < 0.05, respectively) (Figure 8iv). A similar reduction was observed in layers 2/3 after 5 days, but this reduction did not reach significance. In the left Au1, there were significant reductions in median neuronal densities in layer 2/3 after 24 h (by 31%, *p* < 0.05) and layer 4 after 24 h (by 35%, *p* < 0.05) (Figure 8v). Smaller reductions in neuronal density were observed after repeated blasts in layer 4 after 5 days and layers 5/6 after 24 h and 5 days, but these reductions did not reach significance. In the right Au1, reductions were observed in layers 4 and layers 5/6 after 24 h and 5 days in the repeated blast group, but these reductions did not reach significance (Figure 8vi). 

To further map cortical neuronal density after blasts, we investigated the effects of repeated blasts on the retrosplenial cortex (RSC), somatosensory cortex trunk region (S1Tr), and ectorhinal cortex (Ect) (Supplementary Figure S3). Interestingly, the RSC, S1Tr, and Ect appear to be less sensitive to repeated blast injury, with no significant changes in median neuronal density observed in any of the RSC layers investigated (layer 1, layers 2/3/4, and layers 5/6) (Figure S3i,ii). Reductions in neuronal density after repeated blasts in the S1Tr layers 2/3, layer 4, and layers 5/6 (Figure S3iii,iv), as well as in the Ect layers 2/3 and layer 4, occurred (Figure S3v,vi), but these reductions did not reach significance. 

### 3.9. Subcortical Regions Are Differentially Sensitive to Three Repeated Blast Shockwaves

**Figure 9:**
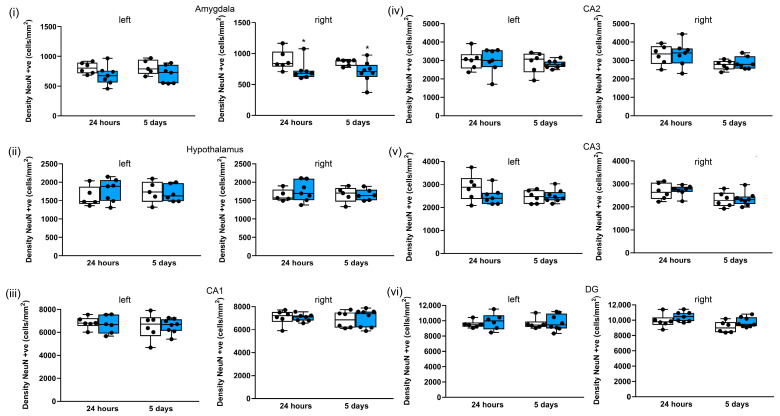
Three repeated shockwaves of 260 kPa result in selective subcortical neuronal loss. Quantification of neuronal cell density in left and right (**i**) amygdala; (**ii**) hypothalamus and hippocampal (**iii**) CA1, (**iv**) CA2, (**v**) CA3, and (**vi**) DG subregions; and in sham (white boxes) in repeated 260-kilopascal blasts (blue boxes). The lines are medians, boxes are interquartile intervals, and whiskers are ranges. * *p* < 0.05 compared to sham; Kruskal–Wallis test with Benjamini–Yekutieli correction. After 24 h, sham n = 6 and blast n = 7; after 5 days, sham n = 6 and blast n = 8.

In the amygdala (Figure 9i), there were significant reductions in neuronal cell density after repeated blasts in the right hemisphere after 24 h and 5 days (by 19%, *p* < 0.05 and 19%, *p* < 0.05, respectively). Similar reductions were observed in the left hemisphere (by 16% and 8%, respectively, at 24 h and 5 days), but these reductions did not reach significance (Figure 9i). In contrast, the hypothalamus (Figure 9ii) appears to be less sensitive to repeated blasts, as there were no significant differences in the median neuronal density following blasts. Similarly, most of the hippocampal subregions appear to be insensitive to repeated blasts (Figure 9iii–v), with the exception of the left CA3 region, where there was a reduction in neuronal density of 17% after 24 h, but this reduction did not reach significance (Figure 9v). There were reductions in the median neuronal density after repeated blasts in the left medial habenular nucleus (8% after 24 h and 5 days) (Figure S4i), and the laterodorsal thalamic nucleus, ventrolateral (17% after 24 h; 15% after 5 days) (Figure S4ii), but these reductions did not reach significance. In contrast there was no change after repeated blasts in the ventral posteromedial thalamic nucleus, ventromedial/ventrolateral thalamic nuclei, or centromedial thalamic nucleus (Figure S4ii–v). 

### 3.10. Exposure to Three Repeated Blast Shockwaves Does Not Result in Astrogliosis in Cortical and Subcortical Regions

We quantified the GFAP-positive area as a measure of astrocyte activation. There were no significant differences in the median GFAP-positive area in any of the cortical or subcortical regions investigated (Supplementary Figures S5 and S6).

## 4. Discussion

### 4.1. Blast Injury Model

Any animal blast TBI model represents a compromise between the multiple heterogeneous (and uncontrolled) factors influencing real-world blast injury and the possibility of carefully controlling and modelling specific aspects of the real-world blast in order to understand the contributions of individual factors. We used a shock tube to generate reproducible shockwaves with a Friedlander waveform typical of a free field explosion. We aimed to model both primary and tertiary blast injuries; therefore, we chose a configuration that included head motion [27,62,63,64,65]. We chose to model head-only blast neurotrauma, in part because the widespread use of modern body armour by military personnel provides effective thoracic protection [66].

There is debate regarding the appropriate scaling parameters between animal models and humans, with mortality-based scaling suggesting that scaling of duration is important [67]. It has been estimated that the relevant scaling factor for blast-wave duration between rodents and humans is around seven-fold; thus, waves with durations of ~1.5 ms in rodents would be representative of exposure of a human to a typical IED blast with a duration of less than 10 ms [66]. However, recent predictions based on blast-induced intracranial pressure measurements in animal models have suggested that the scaling of the overpressure may be more important than the duration [68]. We used shockwaves of peak overpressure of 260 kPa, a duration 1.4 ms, and an impulse of 130 kPa·ms. According to calculations made using the Kingery–Bulmarsh equations [69], an impulse of ~130 kPa·ms corresponds to using 2 kg of TNT at a standoff of 3 to 4 m in free-field conditions. 

### 4.2. Characterisation of Head Kinematics

There has been controversy regarding the relative contribution of the shockwave (primary injury) and acceleration (tertiary injury) to blast neurotrauma. Some evidence suggests that head motion plays the most important role [19,62,63,70], while other evidence suggests that the shockwave alone results in significant injury [26,71]. There are relatively few studies that have characterised head motion in detail, and direct comparisons between studies are complicated because head orientation relative to shockwave is different in each model or different species have been used (e.g., the relative sizes of mice and rats affects the kinematics for a given blast intensity). 

Our model’s head motion described an oval shape similar to the ‘bobble head motion’ described by Goldstein *et al.* [19], and it had a similar maximum velocity. However, in our rat model, maximum accelerations were 4 to 8 times smaller in magnitude than those reported in mice [19,27,70]. Given the similarities in peak velocities, the difference in the accelerations likely reflects the approximately 10-fold greater mass of the rat compared to the mouse. Our accelerations are similar in magnitude to those reported by Budde *et al.* in rats [26].

### 4.3. Repetitive Head-Only Exposure to Blast Shockwaves Results in Unconsciousness and Persistent Neurological and Locomotor Impairments

In contrast to the lack of an acute effect of a single 260-kilopascal blast, exposure to three tightly coupled blasts resulted in prolonged blast-induced unconsciousness. Our observation of blast-induced unconsciousness after three repeated blasts, but not after one blast, is consistent with studies in mice that used whole-body exposure [31]. In addition, unlike the case of single blast exposure, we observed significant neurological impairments at 24 h after repeated blast injury and persistent vestibulomotor impairments, which are consistent with reports in both mice and rats [31,37]. We observed weight loss on days 1–5 following repeated blasts, which is consistent with global neurological and sensorimotor impairments. These functional neurologic and motor impairments were among the primary outcomes considered to assess whether blast exposure induced injury.

### 4.4. Absence of Lung Injury in the Head-Only Blast Model

We aimed to investigate isolated blast injury to the head without the potential confounder of systemic inflammatory responses resulting from blast exposure of the thorax, as there is evidence that systemic inflammation can result in neuroinflammation and injury to the brain [72]. We were careful to position the animals such that only the head was exposed to the shockwave, with the thorax being shielded. The lung, being an air-filled organ, is extremely sensitive to injury from blast [73]. In addition to generating a systemic inflammatory response, injury to the lung could result in reduced oxygen exchange, causing hypoxic brain injury. Hence, in order to ensure that our model did not result in lung injury, we measured peripheral oxygen saturation and carried out lung histology after blast exposure. From a functional perspective, oxygen saturations were normal after blast injury, which is consistent with there being no injury to the lungs, and this outcome was confirmed by normal lung histology. 

### 4.5. Mapping Neuronal Loss after Repetitive Blast across Cortical and Subcortical Brain Regions

**Figure 10:**
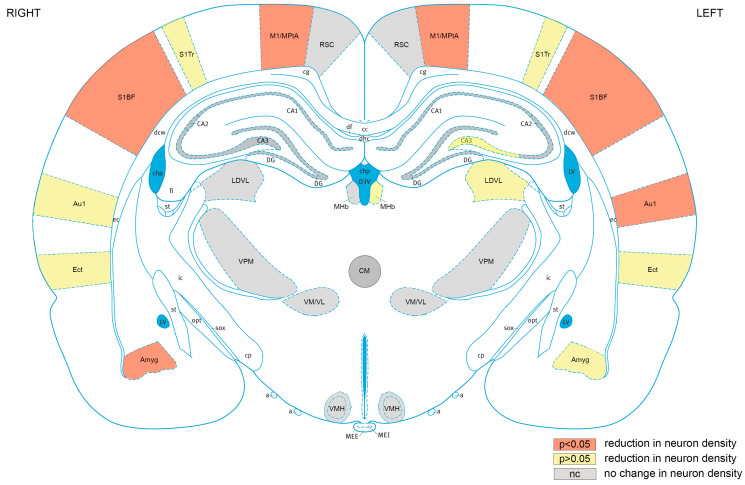
Cortical brain areas are most sensitive to repeated blast TBI. Mapping of regions of interest analysed exhibiting significant (*p* < 0.05) reductions neuronal density (orange), reductions in neuronal density that did not reach significance (yellow), or no change in neuronal density (grey). RSC, retrosplenial cortex; M1/MPtA, motor cortex/medial parietal temporal area; S1Tr, somatosensory cortex trunk area; S1BF, somatosensory cortex, barrel field; Au1, primary auditory cortex; Ect, ectorhinal cortex; Amyg, amygdala; VMH, ventromedial hypothalamic nucleus; VM/VL, ventromedial/ventrolateral thalamic nucleus; VPM, ventral posteromedial thalamic nucleus; LDVL, laterodorsal thalamic nucleus, ventrolateral; MHb, medial habenular nucleus; CA1, hippocampal CA1 region: CA2, hippocampal CA2 region; CA3, hippocampal CA3 region; DG, hippocampal dentate gyrus region. The ventricles are shown in blue. Figure 10 is based on rat brain atlas of Paxinos and Watson [74], and is used with permission.

The regions in which neuronal loss occurred are summarised in Figure 10. The cortex was most sensitive to injury, with significant (*p* < 0.05) neuronal loss occurring in the motor cortex, somatosensory cortex, and auditory cortex, while non-significant reductions in neuronal density occurred in the ectorhinal cortex. The greater sensitivity of cortical areas to repeated blast injury is consistent with our findings of persistent neurological and motor impairments following repeated blasts. Neuronal loss in motor and somatosensory areas associated with motor impairments was our primary histological outcome. We also observed significant neuronal loss in the right amygdala with a non-significant reduction in neuronal density in the left amygdala. Although the amygdala is not a cortical structure, it is located in the outer part of the rat brain, being adjacent to the piriform cortex, and, therefore, physically closer to the cortex than the thalamic subcortical areas. The reason for the sensitivity of these regions to blast is unclear, but it is possible that exposure to the unattenuated shockwave or proximity to changes in tissue density (e.g., skull/CSF/brain parenchyma) may play a role.

Although the blast exposure was lateralised on the right side of head facing the incident shockwave, we observed bilateral neuronal loss in cortical areas. There has been debate as to whether blast shockwaves result in an overall diffuse injury or more focal lateralised injury [19,26]. The bilateral injury perpendicular to the shockwave is consistent with a neuroimaging study of blast injury in rats [26] and modelling of the pressure profiles in rat brain after blasts [75]. Potential mechanisms of bilateral injury development include the shockwave (primary blast injury) traversing both hemispheres, interhemispheric connectivity, acceleration/deceleration (tertiary blast injury) resulting in coup–contrecoup injury, or a combination of all of these factors. In the motor cortex/medial parietal temporal area and the somatosensory cortex barrel field, there was bilateral neuronal loss after blasts compared to uninjured sham group, which is consistent with the neurological impairments and persistent vestibulomotor impairments that we observed following repeated blasts. The primary motor cortex and somatosensory cortex are closely connected and involved in the integration of sensory information and motor function [76]. Vestibular dysfunction is frequent following blast exposure in both military and civilian populations [77,78]. 

Neuronal loss was also observed in the auditory cortex, with greater loss occurring in the left hemisphere. The reason for greater neuronal loss in the contralateral auditory cortex is not clear; nevertheless, it is notable that the right ear is proximal to the shockwave and it is possible that the input to the left auditory cortex from the right ear is attenuated or absent, and this issue merits future investigation. Hearing loss and auditory dysfunction are very common symptoms in blast-exposed veterans [79]. Although we did not measure auditory responses in this study, these findings indicate that the investigation of auditory function in our model may merit further investigation. In contrast to other cortical regions of interest, the retrosplenial cortex was not affected, with no changes observed in neuronal density after repeated blasts. The reasons for the reduced sensitivity of the retrosplenial cortex to blast injury may be related to its anatomical location close to the midline, which may protect it from the incident shockwave and make it less sensitive to acceleration in the *x*-axis. 

Although several *in vivo* studies of blast TBI have observed evidence of neurodegeneration, axonal injury, and tau protein accumulation [19,39,80], relatively few studies have quantified neuronal density after blast injury, and as far as we are aware, there has not been a systematic mapping of neuronal loss across the whole brain. Sajja *et al.* [81] reported significant reduction in averaged NeuN staining in the pre-frontal cortex, nucleus accumbens, and amygdala at 1 month after single whole-body blasts in rats. In a model of focal cranial blasts, Bu *et al.* [82] reported reductions in NeuN-positive staining in the cortex, striatum, and amygdala. In contrast, Elder *et al.* [34], reported no obvious histological changes in Nissl-stained sections of hippocampus and neocortex 4.5 months after repeated blasts in rats, despite PTSD-like behavioural deficits. Although they did not quantify neuronal density using NeuN staining as we did in this study, Wang *et al.* [31] reported FluoroJade B staining (indicating neurodegeneration) in the piriform cortex and amygdala after repeated blasts in mice.

### 4.6. Repetitive Exposure to Blast Shockwaves Does Not Result in Astrogliosis

In order to determine whether the cortical neuronal loss after repeated blasts was associated with astrogliosis, we investigated changes in the GFAP-positive area in the motor cortex/medial parietal temporal area, somatosensory cortex, auditory cortex, and retrosplenial cortex. Interestingly, there were no significant changes in the GFAP-positive area in any of these cortical areas at either the 24-hour or 5-day timepoints, despite significant neuronal loss being observed after repeated blasts in all cortical regions, except the retrosplenial cortex. These findings contrast with those of Sajja *et al.* [81], who observed increases in GFAP-positive staining in the pre-frontal cortex that were associated with neuronal loss and neurodegeneration after a single blast. However, it is worth noting that in addition to examining a different brain area, Sajja *et al.* investigated longer time points of one month and three months after blast. 

A recent study of repeated blast injury in rats observed increases in the GFAP-positive area in specific thalamic nuclei at 4 weeks after blasts that were associated with vestibulomotor deficits [83]. In addition, a study of single blast injury in mice reported bilateral increases in GFAP staining in the thalamus [19]. In contrast, we observed no significant differences in the GFAP-positive area in subcortical regions. However, although it did not reach significance, there was a large increase in the GFAP-positive area in the ventrolateral/ventromedial thalamic nucleus after 24 h. 

Astroglial scarring has been reported as a characteristic of human blast injury [18,84]; it is noteworthy that the majority of human studies have studied chronic time points (months to years after blasts), rather than the acute time points (up to 5 days) that we investigated. Chronic astrogliosis may be the result of late-onset changes that are not manifest at early time points. In support of this viewpoint, in rodent models of blunt TBI, we observed relatively little astrogliosis in the injury group after 24 h [58], but chronic astrogliosis was observed 18 months after injury [85]. 

### 4.7. Limitations and Future Work

Our study shows that repetitive exposure to a subthreshold blast TBI results in acute unconsciousness, persistent neurological and vestibulomotor deficits, and significant neuronal loss in associated brain areas. The functional tests that we used focus on general neurological function and motor behaviour. In addition to neuro-motor deficits, blast injury in humans is associated with cognitive impairments and anxiety/depression [86]. In future studies, we will investigate measures of cognition and anxiety following repeated blasts using our model. We did not investigate RotaRod performance following a single blast; although we cannot be certain that there were no vestibulomotor impairments after a single blast, the fact that there were no blast-induced unconsciousness or impairments in the neuroscore (which includes beam walking, which is a basic test of vestibulomotor function) suggests that it is unlikely that deficits would have been detected using RotaRod. While we observed significant neuronal loss in cortical and subcortical brain areas up to 5 days after injury, we have not investigated the mechanism underlying this finding or looked at chronic timepoints [85]. Future work will address whether mechanisms such as apoptosis or axonal injury underlie the observed neuronal loss, as well as investigate chronic timepoints and other brain regions, such as the cerebellum. Interestingly our study did not observe increased expression of GFAP following blasts, which is in contrast to several other studies [19,81,83]. Increased expression of GFAP has frequently been used as a surrogate measure of astrocyte activation/astrogliosis [87]; however, it is now recognised that astrocyte activation results in a multitude of transcriptomic changes [88]. Future studies using our repeated blast model may reveal whether there is astrocyte activation at longer timepoints, as well as whether it is present at the early timepoints in the absence of increased GFAP expression. 

### 4.8. Translational Relevance

We describe a novel head-only model of repetitive mild blast TBI (mbTBI) incorporating primary blast injury (shockwave) and tertiary blast injury (acceleration/deceleration) that results in acute blast-induced unconsciousness, as well as persistent neurological deficits with selective and significant neuronal loss in motor and somatosensory cortical brain regions correlating with these functional deficits. Our model of repetitive mbTBI demonstrates both acute post-blast unconsciousness (concussion) and lasting functional impairments similar to those that can occur in humans. In addition, the observation that the cortex exhibits neuronal loss after repeated blasts in our model is consistent with neuroimaging studies in blast veterans showing cortical thinning [89,90]. Furthermore, we observed significant neuronal loss in the auditory cortex and the amygdala, which are brain areas associated with functional hearing impairments and anxiety frequently observed after blast injury in humans. We demonstrate that a single mild blast of the same intensity does not result in any observable functional deficits or neuronal loss, indicating that it is the tightly coupled repetitive blast exposure that causes injury. Until recently, most pre-clinical blast injury studies investigated either single blast exposure or repetitive blast with inter-blast intervals of 1 day or more [32,91]. There have been relatively few studies focusing on tightly coupled blast exposures. Such tightly coupled blasts are highly relevant from a military perspective, as they can occur in cases where multiple charges are used in an IED or during ‘breacher’ training or operations. In addition, firing artillery pieces is associated with repetitive mild blast exposure. Our results are consistent with the initial subthreshold blast sensitizing the brain to subsequent subthreshold insults, and with other models of repetitive blast TBI, suggesting that the window of sensitisation ranges from a few minutes to hours [31,51,52,83]. The fact that our model incorporates head movement, such as that occurring in sports concussion, gives wider relevance to the model. Our novel model will be useful in terms of investigating the processes underlying sensitisation by subthreshold intensity insults, as well as further defining the temporal window of vulnerability of the brain; these studies may be relevant to both repetitive mild blast TBI and other types of tightly coupled repetitive brain injury, such as sports-related concussion. 

## Figures and Tables

**Table 1:** Kinematics for exposure to a single mild blast shockwave. Values are mean (SEM), n = 3 animals.

Kinematic Parameter	Maximum Value	At Time
x-displacement	4.0 ± 0.4 cm	12 ms
x-velocity	15.4 ± 1.4 ms^−1^	4 ms
x-acceleration	3806 ± 826 ms^−2^	6.5 ms
y-displacement	3.2 ± 0.5 cm	25 ms
y-velocity	4.9 ± 0.3 ms^−1^	4 ms
y-acceleration	1357 ± 60 ms^−2^	11.7 ms

## Data Availability

The datasets used and/or analysed during the current study are available from the corresponding author on reasonable request.

## Supplementary Materials

The following supporting information can be downloaded at: https://www.mdpi.com/article/10.3390/brainsci13091298/s1: Table S1. Physiologic parameters before and immediately after blast or sham procedure. Figure S1: A single shockwave of 260 kPa does not result in cortical neuronal loss; Figure S2: A single shockwave of 260 kPa does not result in cortical, subcortical or hippocampal neuronal loss; Figure S3: Cortical neuronal loss after repeated 260 kPa blast is selective; Figure S4: Subcortical neuronal loss after repeated 260 kPa blasts is selective; Figure S5: Representative GFAP staining in sham and three repeated 260kPa blast group at 24 hrs in the left hippocampal CA1 subregion. Figure S6: Three repeated shockwaves of 260 kPa do not result in astrogliosis.
