# Supplementary material for: Repetitive, but Not Single, Mild Blast TBI Causes Persistent Neurological Impairments and Selective Cortical Neuronal Loss in Rats
Source: Brain Sci. 2023 Sep 8;13(9):1298. doi: 10.3390/brainsci13091298 (PMC10526452; doi:10.3390/brainsci13091298)
Supplement: Supplementary file 1 [file brainsci-13-01298-s001.zip › brainsci-2561745-supplementary.pdf]

SUPPLEMENTARY INFORMATION

Repetitive, but Not Single, Mild Blast TBI Causes Persistent Neurological Impairments and Selective Cortical Neuronal Loss in Rats

Campos-Pires *et al*

SUPPLEMENTARY MATERIALS AND METHODS

Image analysis ROI dimensions. To perform the neuronal counting in the cortical regions, we used a rectangle with a width of 200 μm placed at the midpoint of the ROI identified from the rat brain atlas [1]. Cortical layers I to VI were identified via human judgement according to the pyramidal cell morphology, soma size, and neuronal density, and the individual layer ROIs were drawn within the 200-micrometre rectangle. We used a circle with a diameter of 600 μm in the amygdala, the centromedial thalamic nucleus, the ventromedial/ventrolateral thalamic nucleus, the ventral posteromedial thalamic nucleus, and the laterodorsal thalamic nucleus ventrolateral; an oval (520 x 670 μm) in the hypothalamus; and a circle with a diameter of 200 μm in the medial habenula nucleus. In the hippocampus, rectangular regions of interest with the following dimensions were centred on the pyramidal cell layers: CA1 (300 x 30 μm); CA2 (200 μm x 70 μm); CA3 (250 x 50 μm); DG (200 μm x 50 μm).

To perform the quantification of astrocyte GFAP-positive area, we used circular regions of interest with diameters of 1300 μm in the primary motor cortex/medial parietal association cortex, primary somatosensory cortex barrel field, primary somatosensory cortex trunk region, and auditory cortex. In the ectorhinal cortex, a circle of 1100 μm in diameter was used, while in the retrosplenial cortex, an oval (1300 x 600 μm) was used. For the amygdala, thalamic nuclei, and hypothalamus, the same regions of interest used to perform NeuN counting were used. In the hippocampus, the outlines of total CA1, CA2, CA3, and DG regions used in astrocyte analysis were drawn for each slice individually using the ImageJ line tool.

Supplementary reference

1. Paxinos G, Watson C: **The Rat Brain in Stereotactic Coordinates**, Compact 6th Edition edn: Academic Press; 2009.

Table S1. Physiologic parameters before and immediately after blast or sham procedure. Values are medians and interquartile intervals. ** $p<0.01$ 'after' versus 'before'; Kruskal–Wallis test with Benjamini–Yekutieli correction or Mann–Whitney test (propofol dose); total number of animals: 1x sham n = 18; 1x blast n = 22, blast; 3x sham n = 22; 3x blast n = 28.

	1 x Sham		1 x Blast	
Parameter	Before	After	Before	After
O_2 saturation (%)	95 [91, 99]	96 [93, 97]	95 [92, 99]	96 [93, 99]
heart rate (min^{-1})	395 [385, 410]	378 [366, 423]	399 [371, 424]	382 [360, 431]
BP systolic (mmHg)	128 [118, 138]	114 [102, 124]	120 [105,150]	120 [100, 134]
BP diastolic (mmHg)	104 [93, 109]	90 [71, 119]	98 [69, 125]	96 [74, 108]
core temperature (°C)	38 [38, 39]	39 [38, 39]	38 [38, 39]	38 [38, 39]
propofol dose (mg/g)	0.024 [0.019, 0.026]		0.022 [0.020, 0.025]	
	**3 x Sham**		**3 x Blast**	
Parameter	Before	After	Before	After
O_2 saturation (%)	98 [97, 99]	99 [97, 99]	97 [96, 99]	98 [96, 99]
heart rate (min^{-1})	405 [372, 416]	367 [326, 392]	365 [354, 402]	367 [337, 393]
BP systolic (mmHg)	132 [130,137]	140 [119, 157]	117 [108, 130]	132 [118, 143]
BP diastolic (mmHg)	107 [104,110]	109 [95, 128]	96 [76,104]	104 [97,122]
core temperature (°C)	39 [38, 39]	37 [36, 37]**	38 [38, 38]	37 [36, 37]**
propofol dose (mg/g)	0.028 [0.023, 0.031]		0.030 [0.024, 0.035]	

Single blast does not result in cortical neuronal loss

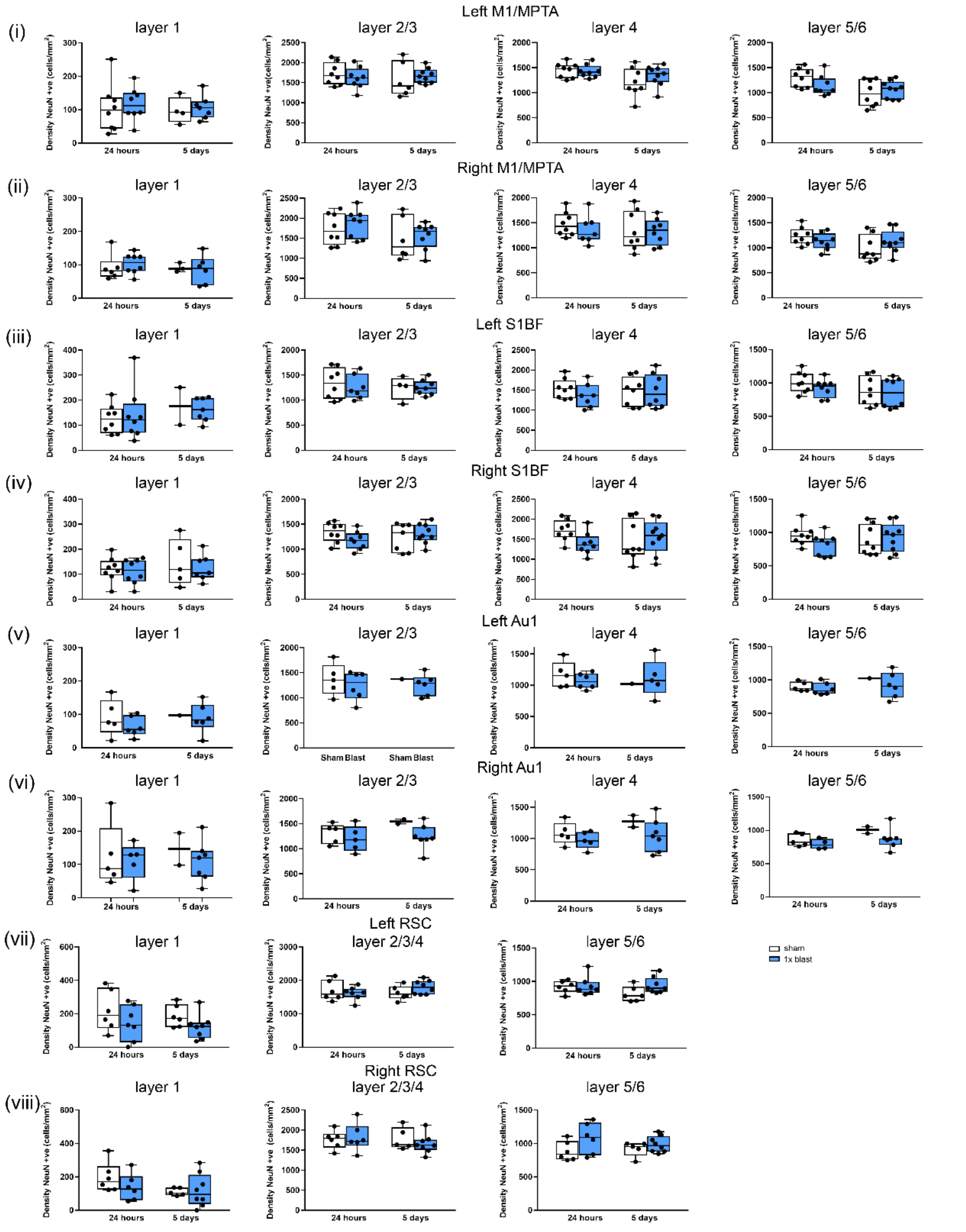

Figure S1. A single shockwave of 260 kPa does not result in cortical neuronal loss**.** Quantification of neuronal cell density of cortical layers from sham (white boxes) and single 260-kilopascal blast (blue boxes) in **(i)** left and **(ii)** right motor/medial parietal association cortex (M1/MPtA), **(iii)** left and right **(iv)** right somatosensory cortex (S1BF), **(v)** left and **(vi)** right auditory cortex (Au1), and **(vii)** left and **(viii)** right retrosplenial cortex (RSC). The lines are medians, boxes are interquartile intervals, and whiskers are ranges. There were no significant differences between sham and blast groups; Kruskal–Wallis test with Benjamini–Yekutieli correction. After 24 h, sham n = 8 and blast n = 8; after 5 days sham n = 8 and blast n = 9. Not all slices included Au1.

Single blast does not result in cortical neuronal loss

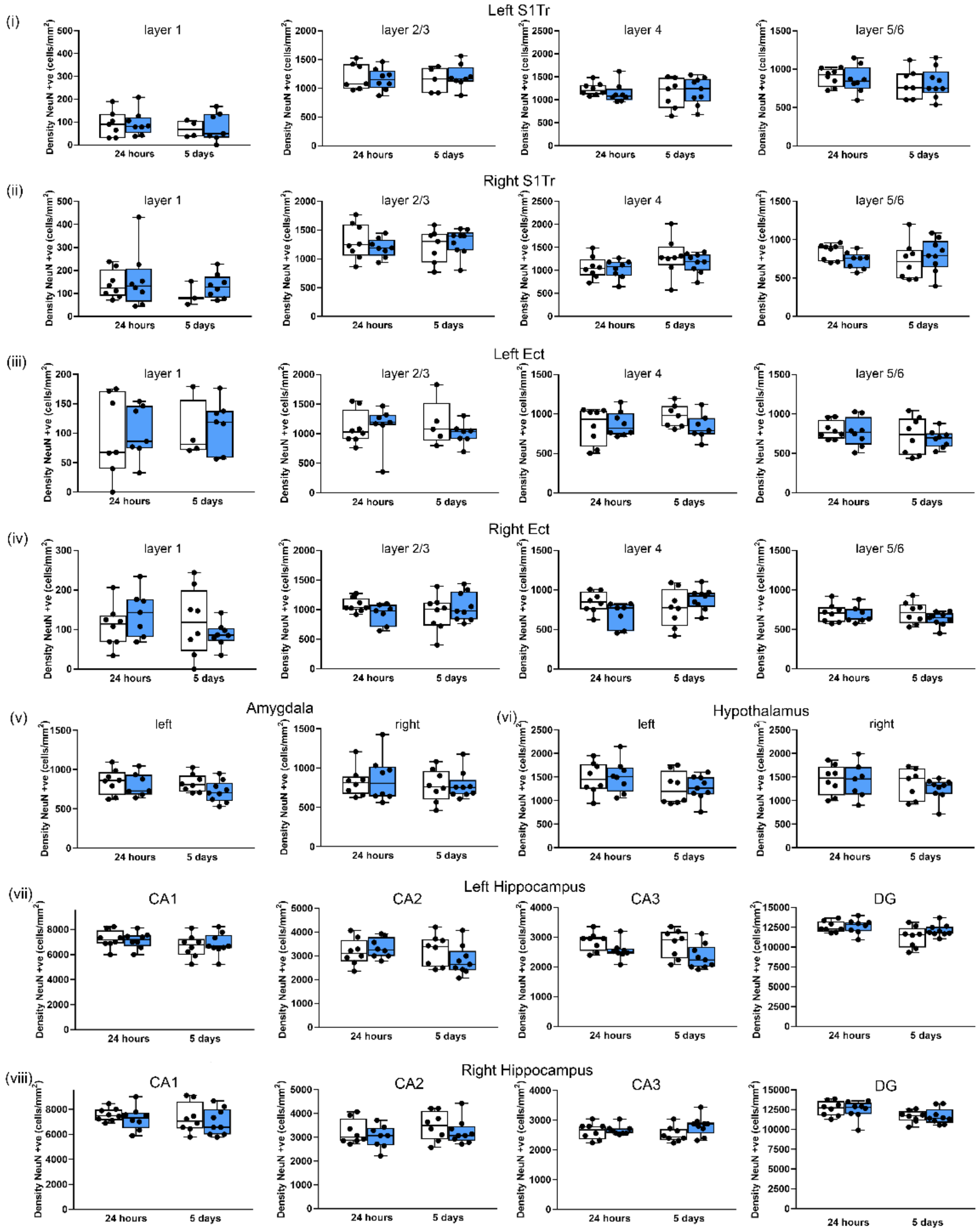

Figure S2. A single shockwave of 260 kPa does not result in cortical, subcortical, or hippocampal neuronal loss**.** Quantification of neuronal cell density of cortical layers from sham (white boxes) and single 260-kilopascal blasts (blue boxes) in **(i)** left and **(ii)** right somatosensory cortex trunk region (S1Tr); **(iii)** left and **(iv)** right ectorhinal cortex (Ect); **(v)** left and right amygdala and **(vi)** left and right hypothalamus; **(vii)** left hippocampal CA1, CA2, CA3, and DG regions; and **(viii)** right hippocampal CA1, CA2, CA3, and DG regions. Lines are medians, boxes are interquartile intervals, and whiskers are ranges. Kruskal–Wallis test with Benjamini–Yekutieli correction. After 24 h, sham n = 8 and blast n = 8; after 5 days, sham n = 8 and blast n = 9.

Repeated blasts result in selective cortical neuronal loss

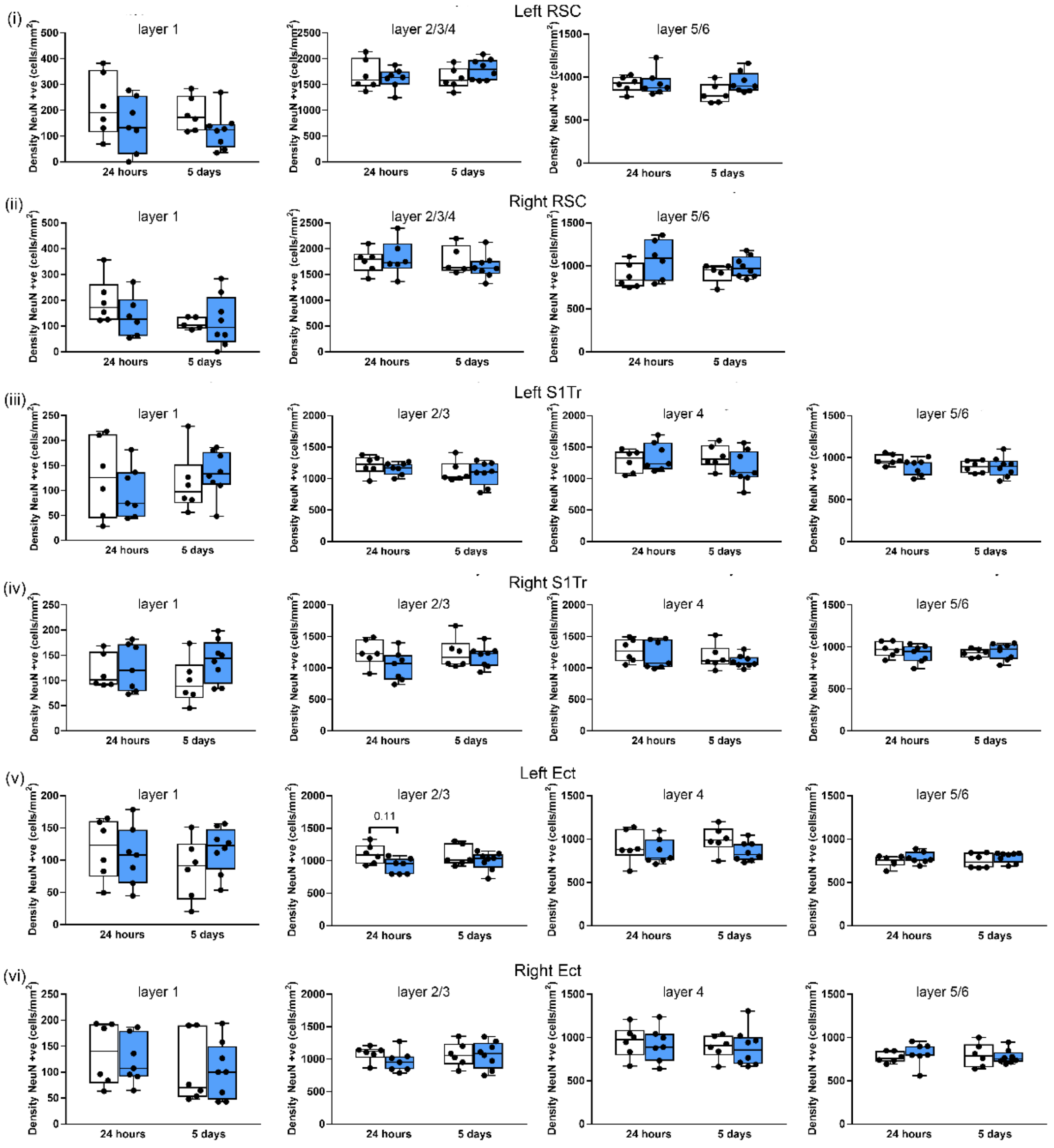

Figure S3. Cortical neuronal loss after repeated 260-kilopascal blasts is selective**.** Quantification of neuronal cell densities of cortical layers from sham (white boxes) and three repeated 260-kilopascal blasts (blue boxes) are shown in **(i)** left and **(ii)** right retrosplenial cortex (RSC), **(iii)** left and **(iv)** somatosensory cortex trunk region (S1Tr), and **(v)** left and **(vi)** right entorhinal cortex (Ect). Lines are medians, boxes are interquartile intervals, and whiskers are ranges. Kruskal–Wallis test with Benjamini–Yekutieli correction. After 24 h, sham n = 6 and blast n = 7; after 5 days, sham n = 6 and blast n = 8.

Repeated blasts result in selective subcortical neuronal loss

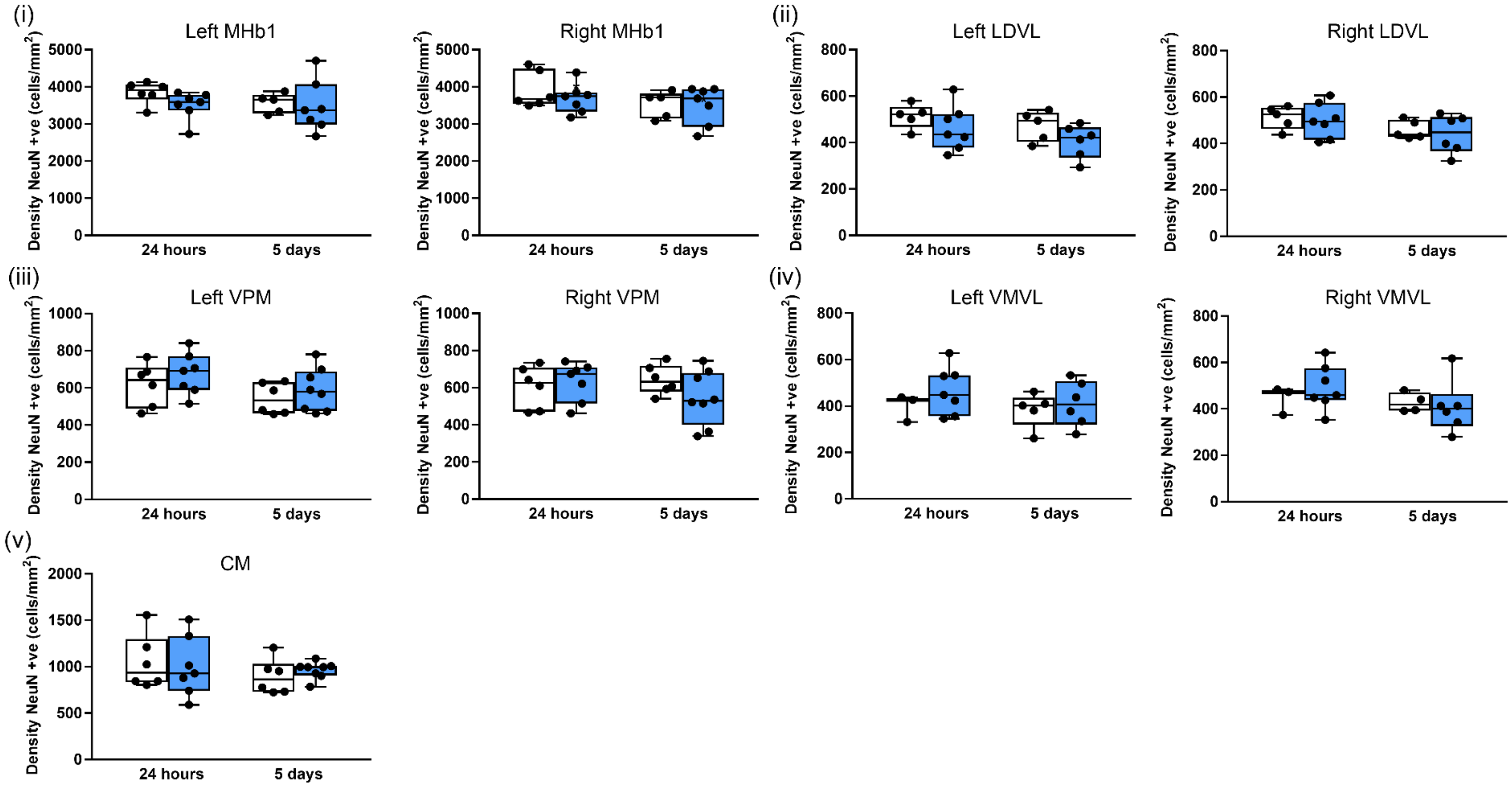

Figure S4. Subcortical neuronal loss after repeated 260-kilopascal blasts is selective**.** Quantification of neuronal cell density of subcortical layers from sham (white boxes) and three repeated 260-kilopascal blasts (blue boxes) in **(i)** left and right medial habenular nuclei, **(ii)** left and right laterodorsal thalamic nucleus, ventrolateral (LDVL), **(iii)** left and right ventral posteromedial thalamic nucleus (VPM), **(iv)** left and right ventromedial/ventrolateral thalamic nucleus (VM/VL), and **(v)** centromedial thalamic nucleus (CM). Lines are medians, boxes are interquartile intervals, and whiskers are ranges. Kruskal–Wallis test with Benjamini–Yekutieli correction. After 24 h, sham n = 6 and blast n = 7; after 5 days, sham n = 6 and blast n = 8.

Repeated blasts do not result in astrogliosis

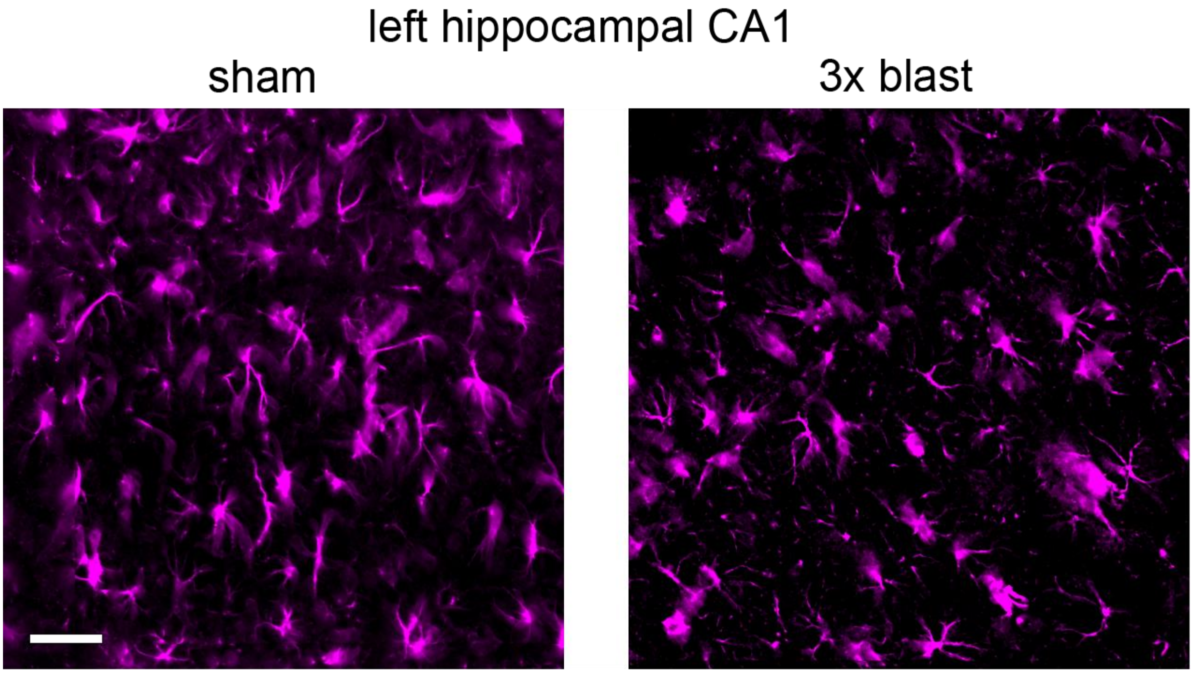

Figure S5. . Representative GFAP staining in sham and three repeated 260-kilopascal blast groups after 24 h in the left hippocampal CA1 subregion. The scale bar is 50 μm.

Repeated blasts do not result in astrogliosis

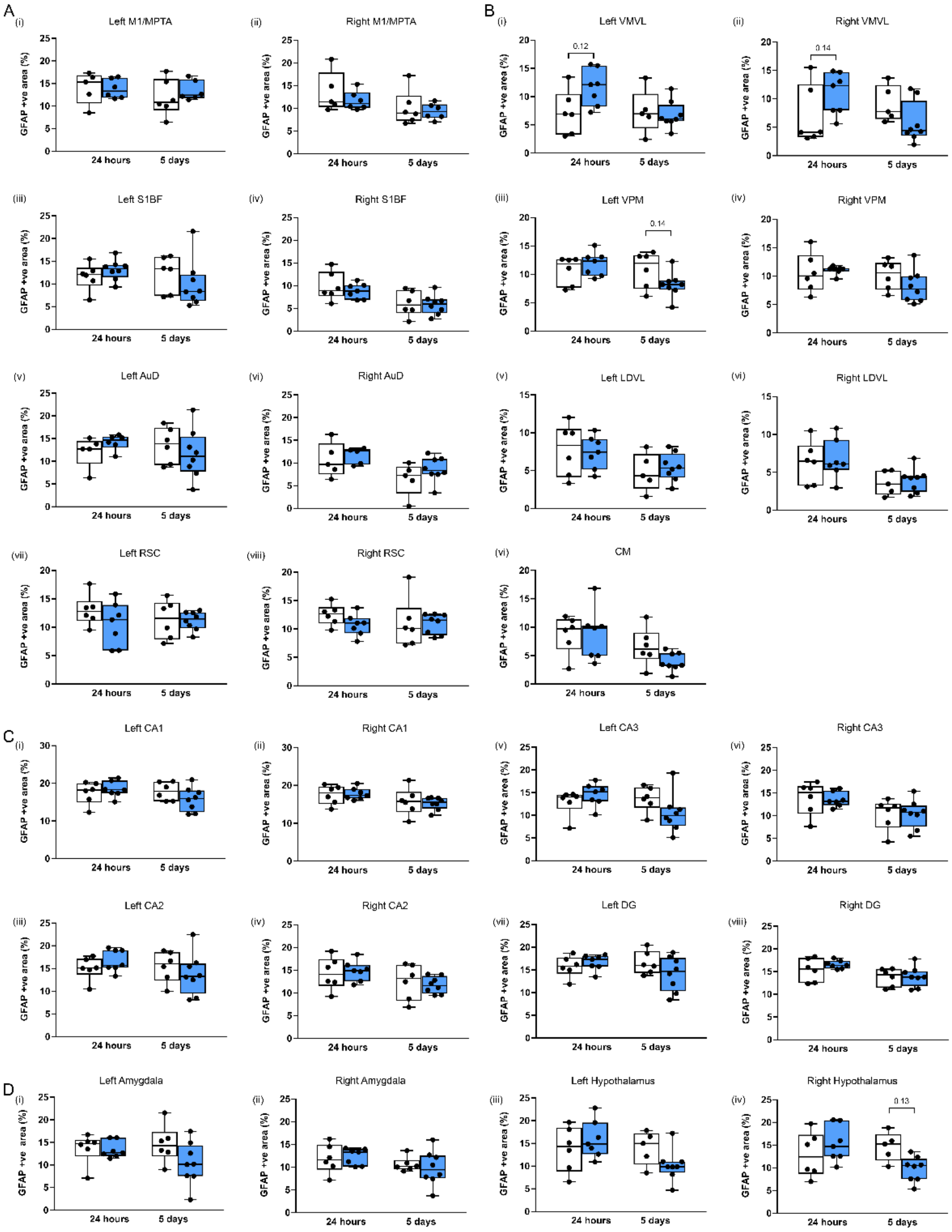

Figure S6. Three repeated shockwaves of 260 kPa do not result in astrogliosis**.** Quantification of GFAP-positive area. **(A)** Cortical areas **(i)** left and **(ii)** right motor/medial parietal association cortex (M1/MPtA), **(iii)** left and **(iv)** right somatosensory cortex (S1BF), **(v)** left and **(vi)** right auditory cortex (Au1), **(vii)** left and right **(viii)** retrosplenial cortex (RSC). **(B)** Thalamic nuclei, **(i)** left and **(ii)** right ventromedial/ventrolateral thalamic nucleus (VM/VL), **(iii)** left and **(iv)** right ventral posteromedial thalamic nucleus (VPM), **(v)** left and **(vi)** right laterodorsal thalamic nucleus, ventrolateral (LDVL), and **(vii)** centromedial thalamic nucleus (CM). **(C)** Hippocampus, **(i)** left and **(ii)** right CA1, **(iii)** left and **(iv)** right CA2, **(v)** left and **(vi)** right CA3, **(vii)** left and right **(viii)** DG. **(D)** **(i)** left and **(ii)** right amygdala, and **(iii)** left and **(iv)** right ventromedial hypothalamus. The lines are medians, boxes are interquartile intervals, and whiskers are ranges. There were no significant differences ($p<0.05$) between sham and blast groups; Kruskal–Wallis test with Benjamini–Yekutieli correction. After 24 h, sham n = 6 and blast n = 7; after 5 days, sham n = 6 and blast n = 8**.**
